# A pre-docking source for the power-law behavior of spontaneous quantal release: application to the analysis of LTP

**DOI:** 10.3389/fncel.2015.00044

**Published:** 2015-02-18

**Authors:** Jacopo Lamanna, Maria G. Signorini, Sergio Cerutti, Antonio Malgaroli

**Affiliations:** ^1^Università Vita-Salute San RaffaeleMilan, Italy; ^2^Neurobiology of Learning Unit, Division of Neuroscience, San Raffaele Scientific InstituteMilan, Italy; ^3^Department of Electronics Information and Bioengineering (DEIB), Politecnico di MilanoMilan, Italy

**Keywords:** spontaneous release, mEPSC, long-term potentiation (LTP), power-law, synapses, hippocampus, allan factor analysis, fractal analysis

## Abstract

In neurons, power-law behavior with different scaling exponents has been reported at many different levels, including fluctuations in membrane potentials, synaptic transmission up to neuronal network dynamics. Unfortunately in most cases the source of this non-linear feature remains controversial. Here we have analyzed the dynamics of spontaneous quantal release at hippocampal synapses and characterized their power-law behavior. While in control conditions a fractal exponent greater than zero was rarely observed, its value was greatly increased by α-latrotoxin (α-LTX), a potent stimulator of spontaneous release, known to act at the very last step of vesicle fusion. Based on computer modeling, we confirmed that at an increase in fusion probability would unmask a pre-docking phenomenon with 1/f structure, where *α* estimated from the release series appears to sense the increase in release probability independently from the number of active sites. In the simplest scenario the pre-docking 1/f process could coincide with the Brownian diffusion of synaptic vesicles. Interestingly, when the effect of long-term potentiation (LTP) was tested, a ~200% long-lasting increase in quantal frequency was accompanied by a significant increase in the scaling exponent. The similarity between the action of LTP and of α-LTX suggests an increased contribution of high release probability sites following the induction of LTP. In conclusion, our results indicate that the source of the synaptic power-law behavior arises before synaptic vesicles dock to the active zone and that the fractal exponent *α* is capable of sensing a change in release probability independently from the number of active sites or synapses.

## Introduction

The study of synaptic transmission has been always hindered by a high level of complexity, arising from the many molecular constituents of the synapse and from the composite dynamics experienced by synaptic vesicles before, during and after their fusion at release sites (Rizo and Südhof, 2002). Undoubtedly, since 1980s, our knowledge about molecular determinants of synaptic vesicles exo-endocytosis has expanded dramatically, while our ability to dissect out vesicles behavior inside the synapse is still very limited. Therefore, it is not surprising for example that a complete understanding of the functional changes underlying all forms of synaptic plasticity is not available yet (Bliss and Lomo, 1973; Bliss and Collingridge, 2013). In the past, the analysis of spontaneous quanta or minis has been one of the most powerful tools to study synaptic transmission. Classically, mini occurrence has been assumed to follow a memory-less Poisson process (Fatt and Katz, 1952). Based on this assumption, variation of the mean Poisson rate of minis has been used to address the state of the presynaptic compartment (Fatt and Katz, 1952; Zucker and Regehr, 2002). Unfortunately, since the beginning of the ‘90s it became clear that at central synapses a variation in mini frequency and/or amplitude is not sufficient to identify the locus of a synaptic change, since both parameters can be altered by pre- and/or postsynaptic phenomena (Edwards, 1991), including variation in the number of functional synaptic units (Isaac et al., 1995; Liao et al., 1995). Because of this, many alternative approaches have been developed to evaluate the occurrence of presynaptic changes following long-term potentiation (LTP) induction (Malgaroli et al., 1995; Ryan et al., 1996; Emptage et al., 2003; Bayazitov et al., 2007; Ratnayaka et al., 2012). Regarding spontaneous synaptic events, one important aspect that has not been fully exploited yet relates to the temporal dynamics of mini occurrences. Even in the absence of a macroscopic change in release probability, when synapses display a steady mini frequency, deviations of spontaneous quantal release from the homogeneous Poisson model might occur. This consideration arises from the well-known complexity of the release machinery and from the presence of a multitude of molecular players that might introduce spatial and temporal correlations in series of quantal releases. In fact, some evidence for deviation from a homogeneous Poisson model has been reported, including multi-exponential mini interval distributions (Abenavoli et al., 2002) and 1/f behavior of the mini rate (Lowen et al., 1997). Therefore, the timing of spontaneous fusions could be important *per se* and its analysis should provide valuable and more direct information on the state of the presynaptic *bouton* and its release machinery. Our results are compatible with previously published evidence suggesting a power-law structure of spontaneous release (Lowen et al., 1997; Takeda et al., 1999). Importantly, here we show that the power-law behavior is greatly enhanced when release probability is augmented. Since we found that also the induction of LTP enhances this power-law behavior of spontaneous quantal release, our findings support the view that LTP must be at least in part sustained by an increase in release probability (Bliss and Collingridge, 2013). We conclude that the spontaneous quantal release echoes a power-law process occurring before synaptic vesicles dock to the active zone, hence the fractal exponent *α* can sense changes in release probability.

## Materials and methods

### Primary cultures of CA3-CA1 neurons

Primary postnatal CA3-CA1 neuronal cultures were prepared from Sprague-Dawley rats (2–5 days old) as previously described (Malgaroli et al., 1995). Research and animal care procedures were approved by our Institutional Animal Care and Use Committee for Good Animal Experimentation, in accordance with the Italian Ministero della Salute code of practice for the care and use of animals for scientific purposes (IACUC number: 576). In brief, P2-P5 rats of both sexes were rapidly decapitated and the CA3-CA1 region of hippocampus dissected out. Dissociated neurons were grown on poly-L-ornithine (10 µg ml^−1^) and Matrigel (1:50 dilution; Becton, Dickinson and Company, New Jersey) coated 35 mm petri dishes (BD Falcon, Becton, Dickinson and Company, New Jersey). Neurons were maintained in a CO^2^ incubator (5% CO^2^, 37°C; Heraeus Instruments GmbH, Hanau, Germany) using a modified minimum essential medium (MEM) with Earle’s salts (Gibco®, Life Technologies) in 5% dialyzed fetal calf serum (Gibco®, Life Technologies). The MEM medium was supplemented with insulin (30 mg l^−1^), biotin (0.1 mg l^−1^), B12 vitamin (1.5 mg l^−1^), L-ascorbic acid (100 mg l^−1^), transferrin (100 mg l^−1^; Calbiochem®, Merck KGaA, Darmstadt, Germany), Glutamax (100 mg l^−1^; Gibco®, Life Technologies), D-glucose (6 g l^−1^), 4-(2-hydroxyethyl)-1-piperazineethanesulfonic acid (HEPES) (3.6 g l^−1^), gentamicin (2 mg l^−1^). Every 3 days one third of the culture medium was replaced with fresh medium supplemented with cytosine β-D-arabinofuranoside (ARA-C; 2.5–5 µM) to prevent excessive glial cells proliferation. If not otherwise indicated, salts and chemicals were obtained from Sigma-Aldrich (Sigma-Aldrich, St. Louis, MO).

### Electrophysiological recordings

CA3-CA1 cultured neurons were used for electrophysiological experiments 10–21 days after plating. All electrophysiological recordings were performed at room temperature (24°C) and neurons were continuously superfused (1–2 ml min^−1^) with a Tyrode solution containing (in mM): 119 NaCl, 5 KCl, 2 CaCl_2_, 2 MgCl_2_, 25 HEPES and 30 D-glucose. This solution was supplemented with the GABA_A_ receptor blocker picrotoxin (PTX; 100 µM), the voltage-gated sodium channel blocker tetrodotoxin (TTX; 0.5–1 µM; Latoxan, Valence, France) and the NMDA receptor blocker D-2-amino-phosphonovalerate (APV; 25 µM; Tocris Cookson, Bristol, UK). Solution osmolarity was adjusted to 305 mOsm and pH to 7.4. Patch pipette electrodes (resistance 5–10 MΩ) were filled with an intracellular solution containing (in mM): 110 D-gluconic acid, 5 MgCl_2_, 10 NaCl, 0.6 Ethylene glycol-bis(2-aminoethylether)-N,N,N′,N′-tetraacetic acid (EGTA), 2 ATP, 0.2 GTP, and 49 HEPES (pH adjusted to 7.2 with CsOH; osmolarity 290 mOsm). Currents were acquired in voltage clamp (holding potential −70 mV; Axopatch 200B amplifier; Axon Instruments, Foster City, CA) using either the standard whole-cell mode or in perforated-patch configuration (amphotericin B, 0.25 µg ml^−1^). Membrane and series resistances were constantly monitored by applying 2–5 mV depolarizing pulses. Recordings with either unstable input/series resistance or with series resistance values above 20 MΩ (whole-cell), 30 MΩ (perforated-patch) were discarded. Current traces were filtered at 2–5 kHz and digitally acquired at 20 kHz using a 16-bit analog-to-digital interface (HEKA ITC-18; HEKA Elektronik, Lambrecht/Pfalz, Germany) controlled by a C/C++ acquisition software developed in house. Miniature currents recorded in our conditions were fully suppressed by application of the AMPA receptor antagonist 6-cyano-7-nitroquinoxaline-2,3-dione (CNQX; 10 µM; Tocris Cookson, Bristol, UK) (data not shown). During recordings, drugs were applied either through the bath perfusion system or via a motorized linear array of 5 glass capillaries (diameter 500 µm) positioned above the cells (complete exchange of solution in about 20 ms). If not otherwise indicated, salts and chemicals were obtained from Sigma-Aldrich (Sigma-Aldrich, St. Louis, MO). In these experiments we selected for recordings postsynaptic cells with a spindle like morphology, reminiscent of the *in situ* CA1 phenotype (Malgaroli and Tsien, 1992).

### Induction of LTP

For LTP experiments, cells were locally perfused through an array of capillary tubes (linear array of 5 glass tubes, tube diameter 500 µm) moved by a custom built fast stepping motor device controlled by our acquisition software. This motorized tubes array was placed above the cell from which minis were recorded and allowed a complete replacement of the extracellular solution in less than 20 ms. Just before LTP induction the array was positioned in place and cells were briefly perfused with a Tyrode solution without APV (30–60 s) supplemented with Glycine (1–10 µM; Sigma-Aldrich, St. Louis, MO). By switching the set of capillary tubes, neurons were briefly exposed to a high potassium solution (a modified Tyrode solution containing 90 mM KCl and 34 mM NaCl, Glycine 1–10 µM, 305 mOsm). The induction protocol consisted of 3 pulses of 200 ms duration with an inter-pulse interval of 5 s. After induction the array was removed from the recording area. Miniature activity was recorded for at least 15–20 min before and 29–84 min after the induction of LTP. The diameter and the position of the glass tubes were chosen to bathe most of the apical and basal dendritic arborizations of neurons. The goal was to obtain a homogenous potentiation of recorded synaptic populations (the center of the tube was positioned at the center of the cell somata). To prevent the induction of LTP, glycine was removed, APV (25 µM) was added to all extracellular solutions and 1,2-Bis(2-aminophenoxy)ethane-N,N,N′,N′-tetraacetic acid (BAPTA; 10 mM; Calbiochem®, Merck KGaA, Darmstadt, Germany) was included in the intracellular recording solution. In BAPTA experiments, after establishing the whole-cell configuration, we always waited 20–30 min before acquiring miniature currents to allow a reliable diffusion of BAPTA into the proximal and distal dendrites.

### Mini detection algorithm and analysis of mini amplitude and rise time

From all the acquired recordings, minis were extracted by means of a custom detection algorithm based on wavelet filtering (Wavelet ToolboxTM of MATLAB®, MathWorks, Inc.). Briefly, current traces were high-pass filtered for baseline removal (*f*_c_ = 2 Hz, 8th order Butterworth filter) and multilevel wavelet decomposition was applied (5 levels, reverse bi-orthogonal spline wavelet). After thresholding the resulting detail coefficients (thresholds: 1.407, 3.955 for level 1 and 2, respectively; 4.285 for levels 3 to 5), a reconstructed signal was obtained. This signal was then used for the actual detection with a fixed threshold (around −3 pA in most experiments). The output was the starting point of mini waveform. For each detected mini, the exact peak location was determined as the local minimum where the amplitude was computed. Peak-amplitude was expressed as *I*_peak_ − *I*_start_. For measurement of rise and decay times, minis were fitted by a bi-exponential function using a Levenberg-Marquardt algorithm. In order to test detection reliability in high frequency conditions, we performed a sensitivity assay based on simulated mini recordings. Control minis (10 min epochs, *n* = 5 experiments) were used to generate distributions of rise and decay time constants, which were fitted by Gaussian probability density functions. A large number of simulated minis (*n* = 10000) were then obtained by random sampling from the latter distributions and used to generate random sequences of *n* = 100 minis at a variable frequency ranging from 10^−0.1^ to 10^2^ (10 different mini frequency values, 10 independent simulations for each frequency value). Zero-mean white noise with standard deviation (SD) equal to 1/3 of mini amplitude was added to these sequences to get a signal to noise ratio (SNR) of 3. The detection algorithm (with fixed detection threshold equal to −1 pA) was then applied to each simulated recording in order to compute the true positive rate (with 0.6 ms tolerance from their exact location). After detection, some α-latrotoxin (α-LTX) experiments were excluded from further analysis because of excessive instantaneous mini frequency (impairing a reliable detection) or strong non-stationary behavior (overall 3/8 α-LTX experiments).

### Quantification of power-law behavior

Based on previous reports (Lowen and Teich, 2005; Lamanna et al., 2012), two methods that proved superior and/or are more suited for quantifying the fractal exponent *α* were selected. These are the periodogram (PG) and Allan factor (AF). Periodogram-based quantification was implemented according to previously published algorithms (Thurner et al., 1997; Lowen and Teich, 2005; Lamanna et al., 2012). The length of the series (i.e., the time of the last release, *L*) is divided in contiguous windows of length *T*. *C* = *L*/*T* series *W*_i_ are then obtained, by further dividing each window in *M* segments of 10 ms (fixed resolution) and counting the number of events falling in each segment. A PG is then computed for each series *W*_i_: $S_{w}\left(f\right)=\frac{1}{M}{\left|W\left(f\right)\right|}^{2}$ (where *W(f)* is the discrete Fourier transform of the count series *W*_i_). Then the mean PG, *S*(*f*), is obtained by averaging all PGs. This count-based PG is an accurate estimate of the power spectral density (PSD) of the point process for the range: 1/*T* - *M*/2*T* Hz, and follows a power-law of the form *S(f)* ≈ 1/*f*^α^ in the low and medium frequency range for fractal-rate point processes (Lowen and Teich, 2005). We estimated *α* by linear least-mean-square regression on doubly logarithmic scale (log_10_(*PG*) *vs.* log_10_(*f*)), excluding *f* = 0 and imposing a fixed cut-off frequency (0.3 Hz). The AF is derived from Allan variance. For a point process, it is computed as: $AF\left(Tau\right)=E\left[{\left(Z_{k+1}-Z_{k}\right)}^{2}\right]/2E\left[Z_{k}\right]$, where Z_k_ is the count series obtained with a counting window of length Tau (Lowen and Teich, 2005). The AF for a fractal point process assumes the power-law form *AF*(*Tau*) ≈ 1 + (*Tau/Tau*_0_)*^α^*, where *Tau*_0_ is the fractal onset time. Estimating of *α* was achieved by linear least-mean-square regression on doubly logarithmic scale (log_10_(*AF*) *vs.* log_10_(*Tau*)) from *Tau*_0_ = 1 s on.

### Statistics

In order to determine the statistical significance of the power-law behavior, we applied a Monte Carlo approach. For each recording segment used for analysis, we constructed the null hypothesis by simulating *n* = 10000 point processes with exponential inter-events interval distribution with mean parameter *λ* = *T*/*N*, where *T* is the length of the segment and *N* is the number of detected minis. Hence, the null hypothesis was represented by a perfectly homogeneous Poisson process of duration and number of events similar to those of experimental data segments and should theoretically result in an estimated *α* = 0. We computed an *α* estimate for each simulation with both PG and AF (*α*_Poiss_), then obtained a *p*-value for each experiment by dividing the number of times *α*_Poiss_ was higher than or equal to the one observed in real data by *n* = 10000. These algorithms for the evaluation of significance were implemented in C/C++ due to the high computational burden.

### Computer simulations

To predict the effects exerted on the value of *α* by altering either the number or release probability of *N* independent release sites, we run simulations based on the hypothesis that the source of power-law behavior is internal to the presynaptic compartment. In brief, we generated at each release site a series of spontaneous fusion events, where inter-events intervals were obtained by the sum of two independent contributions: (i) the first return time of a random walk process; (ii) an exponentially distributed delay. The first contribution relates to the time required by a random walker, moving at 90 nm s^−1^, to get back to the release site (*x* = 0; discrete steps *ds* of constant size, uniformly distributed, *ds* = +/−0.5 nm). For the first round, *x*_0_ was randomly selected from a uniform distribution between 0 and −100 *ds*. After each additional cycle, the walker position was reinitialized imposing *x*_0_ = −0.5 nm. Diverging simulations (i.e., longer than 10^6^ steps) were aborted and a new simulation cycle started. This procedure can be seen as the level crossing by a symmetric random walk where the crossing level is set to zero. This is characterized by a power-law distribution of waiting times (first return times; Ding and Yang, 1995). The second contribution is meant to simulate the distribution of waiting times for fusion of docked vesicles. Based on Poisson formalism, this delay should follow an exponential distribution with mean frequency parameter *λ* (spontaneous fusions rate of docked vesicles). In these simulations we varied either the number *N* of independent release sites, whose minis series are superimposed over time, or the average frequency parameter *λ* common to all sites. *N* ranged between 10 and 100 (with step size 10; 10 values of *N*), while *λ*^−1^ ranged between 0.1 and 10 s (with step size 0.1 s; 100 values of *λ*). For each pair of parameters, we obtained 10 different simulations, from each one these we estimated *α* by PG and AF methods and averaged the resulting *α* values to obtain a single estimation for each condition. The dependance of *α* on the value of *λ* was fitted by a bi-exponential function using non-linear least square fitting. All the algorithms were developed in house using the MATLAB® environment (MathWorks, Inc.).

## Results

### Incidence of power-law behavior at unperturbed synapses

**Figure 1:**
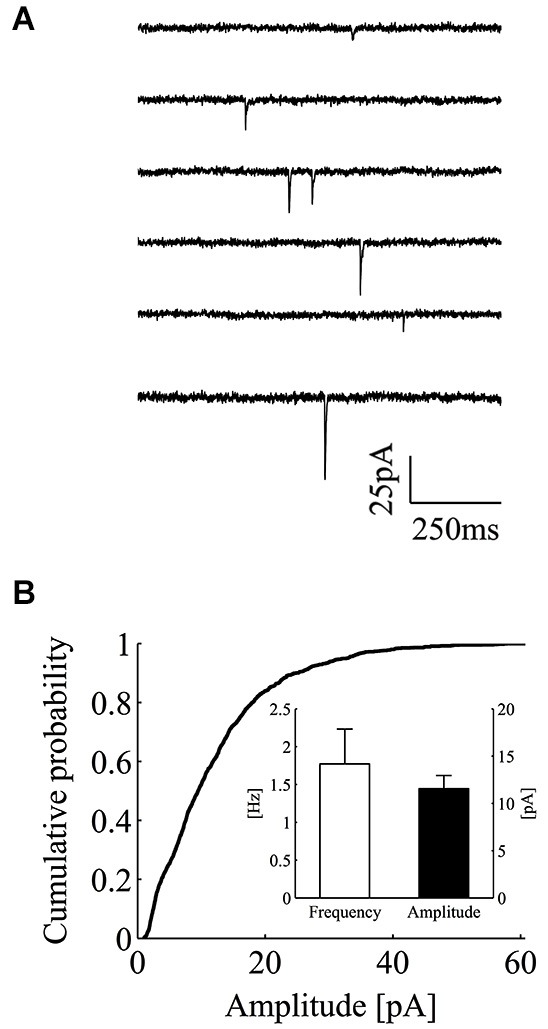
**Whole-cell recordings of minis from CA3-CA1 hippocampal neurons. (A)** Consecutive current traces showing individual excitatory minis in control conditions. Minis were recorded in the presence of TTX (0.5–1 µM), picrotoxin (100 µM) and APV (25 µM). **(B)** Empirical cumulative probability plot for mini peak-amplitudes in one exemplar control experiment. **Inset**, bars plot the average value of mini frequency (white bar; 1.77 ± 0.46 Hz; mean ± SEM; *n* = 15) and peak amplitude (black bar; 11.56 ± 1.39 pA; mean ± SEM; *n* = 15) in the control data set (holding potential = −70 mV).

We recorded miniature excitatory postsynaptic currents (mEPSCs or minis) from CA3-CA1 hippocampal neurons in the whole-cell mode configuration (Figure 1A). In these experiments, neurons were bathed with a Tyrode solution containing tetrodotoxin (0.5–1 µM), the NMDA receptor blocker APV (25 µM) and the non-competitive GABA_A_ receptor blocker picrotoxin (100 µM). Recordings were carried out for extensive periods of time (53 min on average) and the analysis was performed only on those recordings characterized by stable recording conditions. In control, minis occurred at a fairly steady rate with an average frequency of 1.77 ± 0.12 Hz (Figure 1B, inset; mean ± SEM; *n* = 15). Mini amplitudes displayed the classical right skewed distribution, with average amplitude of 11.6 ± 0.3 pA (Figure 1B, inset; mean ± SEM; *n* = 15). In these experimental conditions, the good separation between background noise and mini amplitudes allowed a reliable mini detection even for nearly superimposed miniature events (see Section Materials and methods for details and detection algorithm).

**Figure 2:**
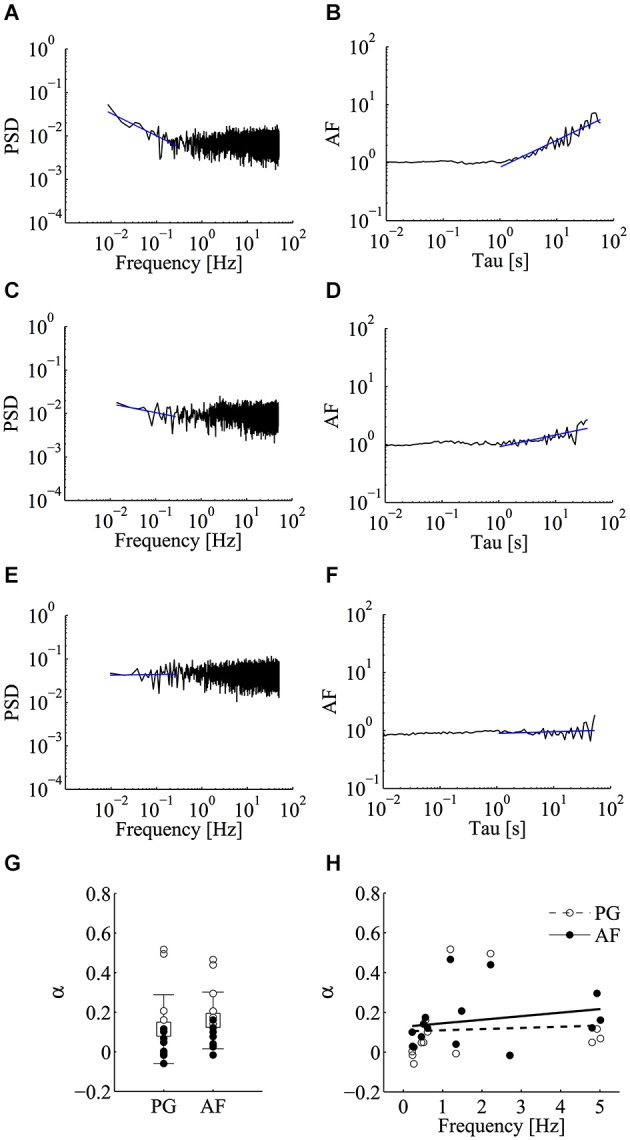
**Low incidence of power-law behavior of miniature activity in control conditions**. Analysis of power-law behavior for the control data set using both power spectral density (PSD) of the estimated mini rate (periodogram, PG) and Allan Factor (AF). **(A–F)** Power-law analysis in three representative recordings using the PG method (left panels) and the AF method (right panels). Blue solid lines refer to fitting of the power-laws to estimate *α* exponent. Few control experiments showed a steep power-law as in panels **A,B**, while in most cases the value of the *α* exponent was either small (panels **C,D**) or close to zero (panels **E,F**). **(G)** Values of *α* estimated using PG and AF on individual control experiments. White and black circles refer to experiments where we could or could not reject the homogeneous Poisson hypothesis, respectively (PG: *p* < 0.05 for 4/15 cases; AF: *p* < 0.05 for 6/15 cases). Average values (boxes) and error bars are shown on the same plots (*α*_PG_ = 0.114 ± 0.174; *α*_AF_ = 0.159 ± 0.143; mean ± SD; *n* = 15). **(H)** Scatter plot of *α* vs. the average mini frequency with linear fitting (PG, white circles; AF, black circles). Each circle refers to an individual control experiment. No significant correlation could be detected between *α* and mini frequency, using both linear and non-linear correlation tests (Pearson correlation test: *p* = 0.83 for *α*_PG_, *p* = 0.42 for *α*_AF_; Spearman correlation test: *p* = 0.15 for *α*_PG_, *p* = 0.13 for *α*_AF_) (see Materials and methods for details).

We began by analyzing the frequency behavior of spontaneous releases in unperturbed control conditions. As depicted in Figure 2, the PSD of the estimated mini rate in some cases diverged from the expected flat spectrum characteristic of a homogeneous Poisson process, with a decrement or scaling of the PSD as frequency increases according to a 1/*f^α^* power-law (Figure 2A). This power-law feature was confirmed by computing the AF, which scaled with time window Tau resulting in an *α* exponent similar to that obtained by PG (Figure 2B; same data set as Figure 2A). Such a fairly clear power-law relation was not seen in the majority of our control experiments, with *α* values either small (Figures 2C,D) or close to zero (Figures 2E,F). In order to evaluate in a more rigorous manner if the *α* values found in control conditions indeed differed from those expected from the null homogeneous Poisson hypothesis and to compute the corresponding *p*-values, we run a set of Monte Carlo simulations. From each experiment, we derived a collection of Poisson realizations (*n* = 10000) with sample size and mean frequency identical to those found in each experiment. Each realization was analyzed by AF and PG methods and *α* was computed. Based on this analysis we could compute a *p*-value for each experiment (see Section Materials and methods). The conclusion is that in control conditions in just 4 out of 15 cases we could find *α* values significantly greater than zero (i.e., rejecting the homogeneous Poisson hypothesis), for both PG and AF (*α*_PG_ = 0.114 ± 0.174; *α*_AF_ = 0.159 ± 0.143; mean ± SD; *n* = 15; *p* < 0.05 for 4/15 cases with PG; *p* < 0.05 for 6/15 cases with AF; Figure 2G). We wondered if this low incidence, which is in contrast with previously published reports (Lowen et al., 1997; Takeda et al., 1999), could somehow relate to some differences in the synaptic state, possibly the synaptic release regime, i.e., spontaneous release probability.

As shown in Figure 2H, in the control dataset we tested for this hypothesis by evaluating the presence of correlation between *α* and the average mini frequency. No clear correlation could be detected with both linear and non-linear correlation tests (Figure 2H; Pearson correlation test: *p* = 0.83 for *α*_PG_, *p* = 0.42 for *α*_AF_; Spearman correlation test: *p* = 0.15 for *α*_PG_, *p* = 0.13 for *α*_AF_). Clearly, the average mini frequency is not *per se* a reliable index of release probability since on one hand relates to the distribution of spontaneous release probabilities at active synapses, but on the other hand also senses the number of active units, a number that is expected to be highly variable among different neurons (Ariel et al., 2013).

### Enhancement of power-law behavior by α-latrotoxin

**Figure 3:**
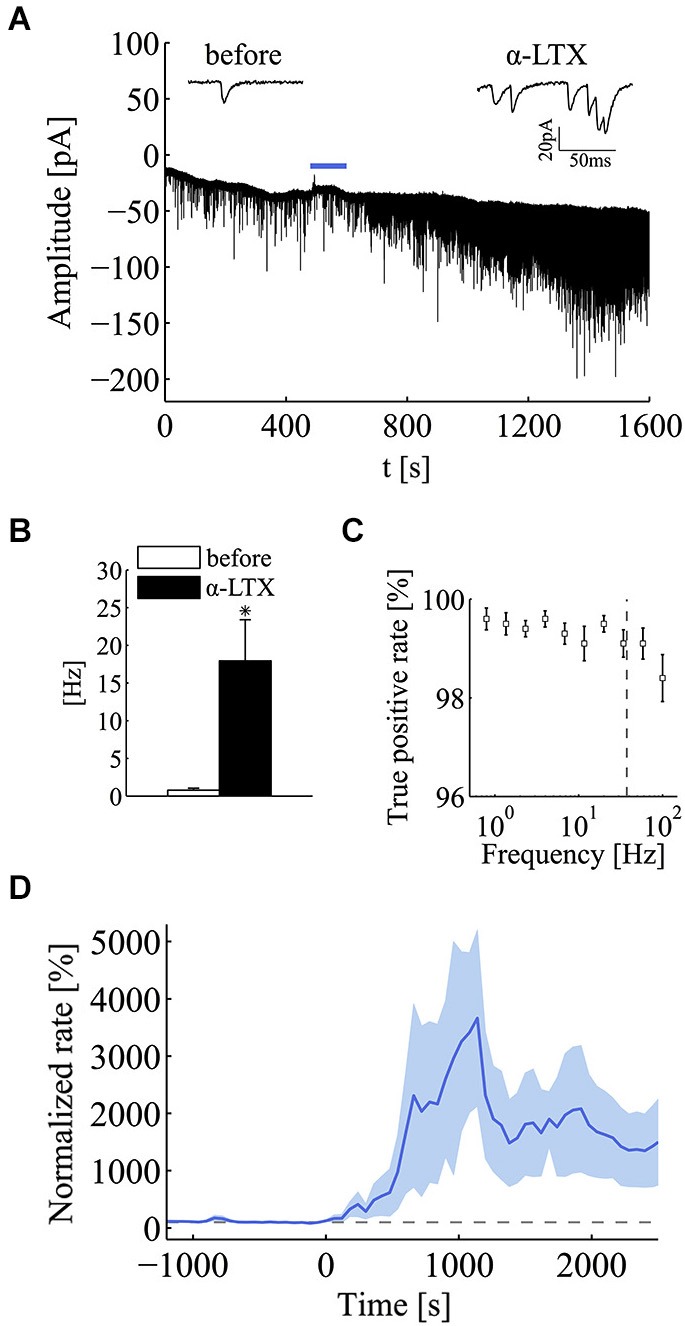
**α-latrotoxin (α-LTX) greatly enhances mini frequency at hippocampal synapses. (A)** Compressed current trace from one exemplar experiment to illustrate the large effect on mini occurrence exerted by α-latrotoxin (α-LTX; 0.5–1 nM). The blue bar refers to the application period of the α-LTX. For the sake of clarity, input-impedance tests and toxin application artifacts were removed from the current trace. Two short sweeps from the same experiments, before and after the application of α-LTX, are shown over the graph. **(B)** Bars plot mini frequency before and after the application of α-LTX (before, white bar, 0.77 ± 0.28 Hz; α-LTX, black bar, 17.95 ± 5.45 Hz; mean ± SEM; *n* = 5; *p < 0.05; Wilcoxon signed-rank test, one tail). **(C)** True positive rate (sensitivity) of the mini detection algorithm over the set of simulated recordings for different values of mini frequencies ranging from 10^−1^ to 10^2^. Sensitivity is always greater than 99.1% for frequencies up to the maximum value observed α-LTX treatment (dashed gray line), confirming the reliability of the algorithm for high mini-rate regimes. **(D)** Normalized mini frequency (thick blue line) averaged across a set of α-LTX experiments (α-LTX application at time = 0 s; frequency bin, 60 s; normalization by the median mini frequency before application). Pale-blue areas above and below the mean frequency refer to standard errors for each bin (see Section Materials and methods for details).

To test more rigorously for the effect of the quantal release regime on the emergence of power-law behavior, we applied α-latrotoxin (α-LTX; 0.5–1 nM), a neurotoxin known to promote a selective increase in spontaneous synaptic vesicle exocytosis (Südhof, 2001). This toxin is known to act at the very last step of exocytosis, in fact its effects are seen even in the absence of extracellular calcium (Capogna et al., 1996) (data not shown). As expected, the application of α-LTX greatly promoted the occurrence of quanta with a large increase in mini frequency (Figure 3A). In this set of experiments, following the application of α-LTX, the average quantal rate increased from 0.77 ± 0.28 Hz to 17.95 ± 5.45 Hz (mean ± SEM; *n* = 5; *p* < 0.05; Wilcoxon signed-rank test, one tail; Figure 3B). To be certain that in these high frequency conditions our detection algorithm could produce a reliable detection of minis, we tested its performance on simulated traces. Based on these simulations we concluded that the detection algorithm was very reliable and could identify minis always with a true positive rate above 99% in the range of frequencies encountered in the experiments here presented (*n* = 10 simulations for each frequency value) (Figure 3C). In the same set of simulations, the specificity of the detection algorithm was found to be around 100% (false positive rate < 1.5 × 10^−3^).

**Figure 4:**
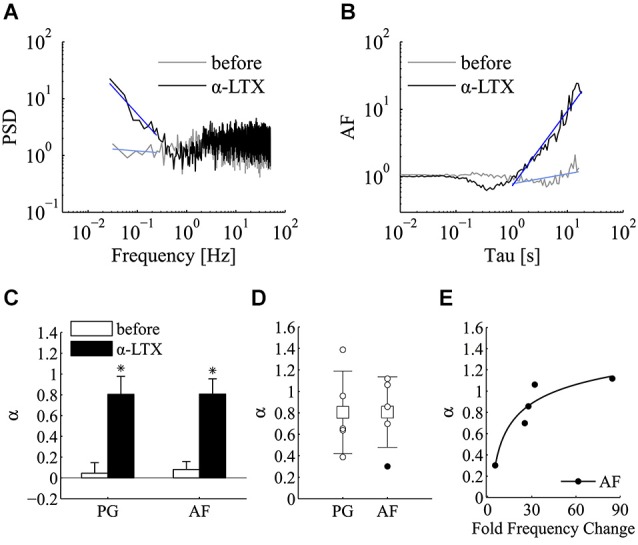
**α-latrotoxin greatly enhances the incidence of power-law behavior of miniature activity**. Analysis of power-law behavior for the α-LTX data set using both PSD of the estimated mini rate (periodogram, PG) and AF. **(A)** Periodogram (PG) before (gray line) and after (black line) the application of α-LTX. The blue lines refer to the power-law fitting to estimate *α* value. **(B)** Same experiment and same experimental epochs as in panel A analyzed by the AF. Line colors and notation as in panel **(A)**. In both panels **(A,B)**, a very clear slope change is detected following α-LTX application. To improve comparisons, PG and AF plots presented in panel **(A,B)** are normalized for the value at *f* = 1 Hz and Tau = 1 s respectively. **(C)** Bars plot the average estimates for *α* measured with both PG and AF methods before (white bars) and after (black bars) the application of α-LTX (*n* = 5 experiments; before *α*_PG_ = 0.046 ± 0.101, α-LTX *α*_PG_ = 0.805 ± 0.171; before *α*_AF_ = 0.081 ± 0.078, 0.143, α-LTX *α*_AF_ = 0.807 ± 0.147; mean ± SEM; *p < 0.05; Wilcoxon signed-rank test, one tail). Note the large increase in the average *α* value following the application of α-LTX. **(D)** Values of *α* estimated using PG and AF on individual α-LTX experiments. White and black circles refer to experiments where we could or could not reject the homogeneous Poisson hypothesis, respectively (PG: *p* < 0.05 for 5/5 cases; AF: *p* < 0.05 for 4/5 cases; see section Materials and methods for statistical testing). Average values (boxes) and error bars are shown on the same plots (α-LTX *α*_PG_ = 0.805 ± 0.383; α-LTX *α*_AF_ = 0.807 ± 0.329; mean ± SD; n = 5). **(E)** Scatter plot of *α* measured by the AF method vs. the average fold change in mini frequency induced by α-LTX (34.9 +/– 29.6 fold; mean ± SD; *n* = 5). Each black circle refers to an individual α-LTX experiment. A non-linear correlation could be detected (*p* = 0.017, Spearman correlation test). The black line is a fitting with a three parameters power function (*y* = *ax^b^* + *c*) (see Section Results for details).

Regarding the effect α-LTX on mini frequency, its initiation and magnitude varied across different cells but in all cases it was found to be long lasting (Figure 3D). After a variable length of time from its application (20–40 min), mini frequency reached a stable level where the power-law analysis was carried out. Interestingly, both PSD and AF plots revealed a very clear power-law behavior following the application of α-LTX (Figures 4A,B), a result that was not sensitive to a shift of the analysis window. As summarized in Figure 4C, the incidence and the magnitude of the power-law behavior were greatly increased by α-LTX. Regarding the magnitude, in all α-LTX experiments the value of the *α* exponent measured with both PG and AF methods was significantly increased following the application of the presynaptic toxin (*n* = 5; before *α*_PG_ = 0.046 ± 0.101, α-LTX *α*_PG_ = 0.805 ± 0.171; before *α*_AF_ = 0.081 ± 0.078, 0.143, α-LTX *α*_AF_ = 0.807 ± 0.147; mean ± SEM; *p* < 0.05; Wilcoxon signed-rank test, one tail). As for control experiments, we run Monte Carlo simulations to evaluate the significance of every estimated experimental *α* exponent found in the presence of α–LTX. With α–LTX, in 4 out of 5 experiments the *α* exponent was found above the 95% confidence limit with both PG and AF methods (Figure 4D; *p* < 0.05 in 5/5 cases with PG; *p* < 0.05 for 4/5 cases with AF). In these experiments, thanks to the large fold change in mini frequency, a correlation between *α* and the fold change in mini frequency by α–LTX could be extracted (Figure 4E; AF method *p* < 0.05, Spearman correlation test).

### A pre-docking source for the synaptic power-law behavior of mini occurrence

Since α-LTX is known to speed up quanta exocytosis by acting at the very last step of the release process, we could postulate that the 1/f behavior originates from some early event preceding or driving the vesicle docking to the active zone. To evaluate the theoretical consistency of this hypothesis, we simulated sequences of docking events following a 1/f process. Downstream this step we placed the spontaneous fusion phase whose timing followed an exponential distribution. In these simulations, we varied both the spontaneous release rate *λ* and the number of release units *N* (100 different values of *λ* ranging from 0.1 to 10 s^−1^; 10 different values of *N* ranging from 10 to 100; *n* = 10 independent simulations for every value of *λ* and *N*; see Materials and methods for the simulation algorithm) and then estimated the value of the fractal exponent *α* by PG and AF methods.

**Figure 5:**
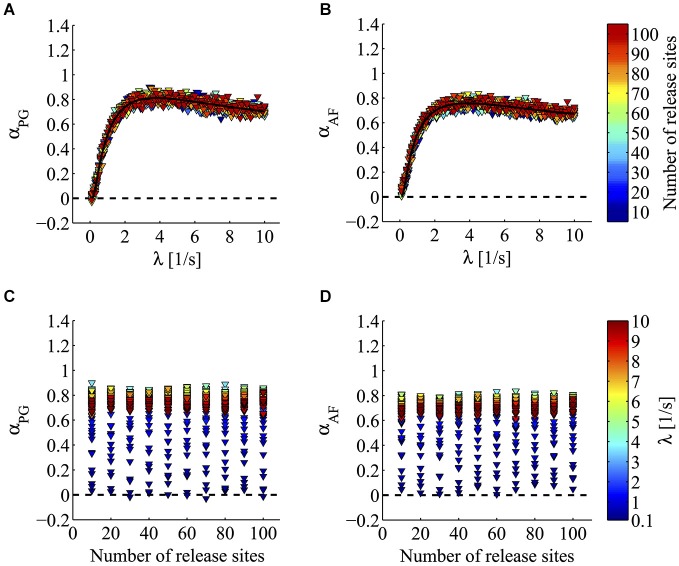
**Computer simulations of minis according to a pre-docking 1/f hypothesis**. Power-law analysis of minis generated according to 1/*f^α^* docking process and a spontaneous fusion phase whose timing followed an exponential distribution (mean parameter 1/*λ*). **(A,B)** Triangles plot *α* values estimated by PG (**A** panel) and AF (**B** panel) from simulated minis series. Triangles with the same color refer to simulations where *α* was computed for a fixed number of release sites (*N*; see lookup table on the right) while varying *λ*. Notice how increasing *λ* strongly enhances the value of *α* estimated by both PG and AF methods (for all *N*, *λ* = 0.1–8.7 s^−1^, *p* < 0.01 for *α*_PG_ and *α*_AF_; Spearman correlation test). The fitted line shows an asymptotic growth profile reminiscent of what found in living synapses for α-LTX experiments (Figure 4E). **(C,D)** Triangles with the same color refer to simulations where *α* was computed for a fixed value of *λ* (see lookup table on the right) while varying the number of release sites *N*. *α* values were estimated by PG (**C** panel) and AF (**D** panel). Notice how despite the very large increase in the number of sites *N*, in these simulations no clear sign of correlation between *α* and *N* could be found (in 95/100, *p* ≥ 0.05 for *α*_PG_; in 89/100 *p* ≥ 0.05 for *α*_AF_; Spearman correlation test).

Figure 5 illustrates the results of this analysis. In this figure, the value of the fractal exponent *α* is plotted as a function of the simulation parameters *λ* and *N*. As illustrated in Figures 5A,B, when the output rate or spontaneous release probability* λ* varied, a steep increase in *α* was detected. In this set of simulations *α* was found to rise from a value around 0 to a plateau value around 1 (*α*_PG_ from −0.03 to 0.9; *α*_AF_ from 0.01 to 0.84; Figures 5A,B). A positive correlation between *α* and *λ* was clearly found for all *N* values (*λ* range analyzed between 0.1–8.7 s^−1^; *p* < 0.01 for *α*_PG_ and *α*_AF_; Spearman correlation test). The relationship between *λ* and *α* was well fitted by a bi-exponential function (*R*^2^ = 0.9633 for PG; *R*^2^ = 0.8544 for AF). This asymptotic growth profile was highly reminiscent of the behavior seen with α-LTX (Figure 4E). On the contrary, as depicted in Figures 5C,D, the 10-fold variation in the number of release units *N* did not produce any clear change in the estimate of the fractal exponent *α* (increasing *N* from 10 to 100 produced a mean increase in *α* of just 0.01 for *α*_PG_ and 0.03 for *α*_AF_). Just to provide a numerical comparison with the very profound effect seen by changing *λ*, a similar 10-fold change in the latter parameter (*λ* from 0.1 to 1 s^−1^) produced a half-maximal increase in (mean value of *α* increase 0.49 for *α*_PG_ and 0.46 for *α*_AF_). Furthermore, in most individual sets of simulations with a fixed *λ* value, no sign of correlation between *α* and *N* could be found (95/100, *p* ≥ 0.05, *α*_PG_; 89/100, *p* ≥ 0.05, *α*_AF_; Spearman correlation test; Figures 5C,D). Based on this, we can conclude that *α*, i.e., the degree of synaptic 1/f behavior, can sense a change in release probability independently from the number of active sites and/or synapses.

### Enhancement of power-law behavior by LTP

**Figure 6:**
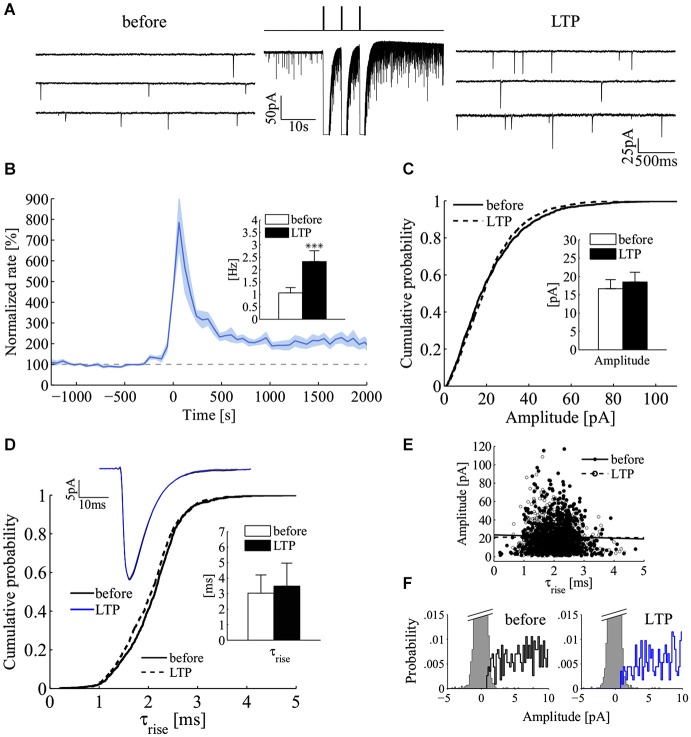
**Increase in mini frequency following the induction of long-term potentiation at hippocampal synapses. (A)** Induction of mini frequency LTP by three short pulses of a high-KCl solution for a representative experiment. Sequential sweeps refer to a control epoch (before, left) and after LTP induction (LTP, right). The compressed current trace in the middle illustrates the LTP induction protocol with the three brief transient responses to the high KCl depolarizing solution (3 pulses of 200 ms, 5 s inter-pulses; isosmotic 90 mM KCl solution). Note the clear increase in mini frequency. **(B)** Normalized mini frequency (thick blue line) averaged across the all set of LTP experiments (LTP induction at time = 0 s; frequency bin, 60 s; normalization by the frequency median value before induction; *n* = 16). Pale-blue areas above and below the mean frequency refer to standard errors for each bin. **Inset**: bars plot mini frequency before and after induction of LTP (before, white bar, 1.06 ± 0.21 Hz; LTP, black bar, 2.32 ± 0.44 Hz; mean ± SEM; *n* = 16; ***p < 0.001; Wilcoxon signed-rank test, one tail). **(C)** Cumulative distributions to compare mini amplitude during a 10 min control period (solid line, before; *n* = 1000 minis) and 20–25 min after induction of LTP (dashed line, LTP; *n* = 1000 minis). **Inset**: bars plot the average mini amplitude in the LTP data set (*n* = 11 experiments; before, white bar, 16.48 ± 2.70 pA; LTP, black bar, 18.5 ± 2.93 pA; mean ± SEM; *p* = 0.44; Wilcoxon signed-rank test, two tails). **(D)** Cumulative distributions to compare miniature rise times before (solid line, before) and after LTP (dashed line, LTP; same experiment and epochs as in panel **C**). **Top Inset**: ensemble average of miniature currents from the same experiment (black line, before; blue line, LTP; averages of *n* = 1000 minis). **Bottom Inset**: bars plot the average miniature rise time before and after the induction of LTP (*n* = 11 experiments; same experiments as in the inset of panel **(C)**; before, white bar, 3.2 ± 1.3 ms; LTP, black bar, 3.6 ± 1.6 ms; mean ± SEM; *p* = 0.41; Wilcoxon signed-rank test, two tails). **(E)** Plot of mini amplitude vs. rise time before and after LTP induction (before, black circles; LTP, white circles; *n* = 1000 minis in both conditions; same experiment as in panels **C,D**). **(F)** Distributions of mini amplitudes and current noise before (left) and after LTP induction (right; same experiment as in panel **C**).

Because cultured hippocampal neurons undergo LTP (Bekkers and Stevens, 1990; Malgaroli and Tsien, 1992; Malgaroli et al., 1995; Ryan et al., 1996; Fitzjohn et al., 2001; Ratnayaka et al., 2012), we tested for the effect of LTP induction on the power-law behavior of spontaneous release. We induced LTP by triggering brief episodes of glutamate exocytosis using very brief applications of a high K^+^ depolarizing solution which was locally applied (Figure 6A; see Methods) (Noel and Malgaroli, 1994; Fitzjohn et al., 2001). Consistently with previous results, LTP induction was characterized by a long-lasting change in quantal frequency, maintained for the duration of the recordings (up to 84 min). The induction procedure raised the frequency of miniature events in 16/17 cells (Figure 6B). Mini frequency increased 2.2 fold above control (before, 1.06 ± 0.21 Hz, LTP 2.32 ± 0.44 Hz, 20–30 min after induction; mean ± SEM; *n* = 16; *p* < 0.001; Wilcoxon signed-rank test, one tail; Figure 6B, inset). In these experiments, no significant change in mini amplitude could be detected. Figure 6C compares mini amplitude distribution in a control period (solid line) with that seen after induction of LTP (dashed line). The two amplitude distributions showed the typical large dispersion of mini amplitudes but a similar shape. On average, there was no significant difference in mean mini amplitude (before 16.5 ± 2.7 pA, LTP 18.5 ± 2.9; mean ± SEM; *n* = 11; *p* = 0.28; Wilcoxon signed-rank test, two tails; experiments characterized by low noise and optimal access resistance). These results, which are relevant to the site of expression of LTP, also indicate that the induction protocol here used is likely to affect homogeneously the recorded synaptic population (see Methods for details about the perfusion protocol). To focus more directly on this issue, we compared mini kinetics before and after induction (Figures 6D,E). Figure 6D illustrates that the rise time distribution and the average mini waveform before and after the induction of LTP match almost perfectly. Similarly, the relationship between amplitude and raise kinetics were left unchanged by LTP induction (Figure 6E). On average, in the same set of experiments used for mini amplitude analysis (Figure 6C), no significant change in miniature rise and decay times was indeed observed following the induction of LTP (τ_rise_ before 3.2 ± 1.3 ms, LTP 3.6 ± 1.6 ms; τ_decay_ before 36.3 ± 11 ms, LTP 39 ± 11 ms; *n* = 11 experiments; mean ± SEM; *p* = 0.41 and *p* = 0.73 respectively; Wilcoxon signed-rank test, two tails) (Figure 6D, inset). These results and the good separation between noise and mini amplitude distributions (Figure 6F) suggest that the here observed change in frequency cannot be accounted by a postsynaptic enhancement of synapses located on distal dendrites, which before LTP produced minis so small to be undetected. Overall these results strongly suggest a homogeneous potentiation of the synaptic population which can only be expressed either by a pure presynaptic change or by the recruitment of silent synapses (Isaac et al., 1995; Liao et al., 1995).

**Figure 7:**
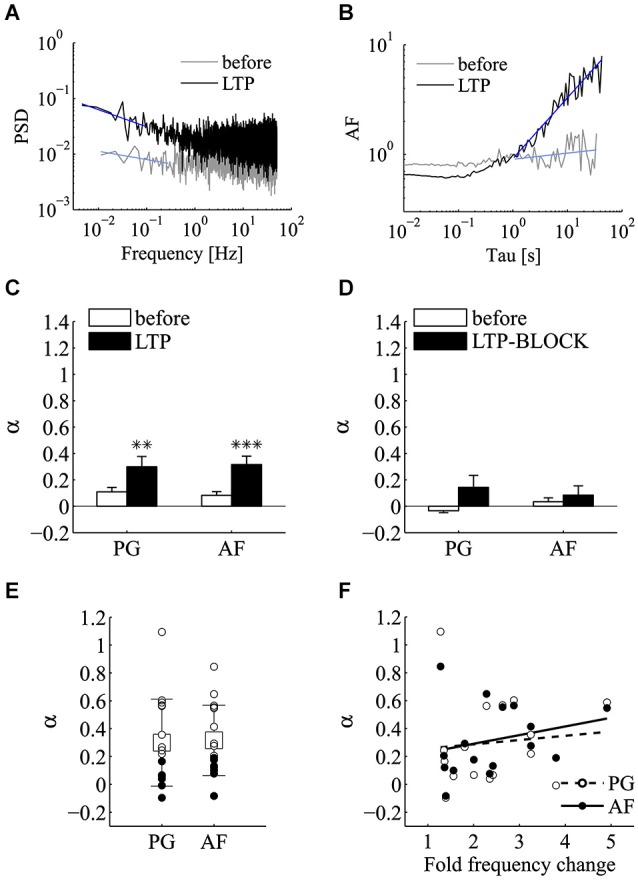
**LTP significantly enhances the incidence of power-law behavior of miniature activity**. Analysis of power-law behavior for the LTP data set using both PSD of the estimated mini rate (periodogram, PG) and AF. **(A)** Periodogram (PG) before (gray line) and after (black line) the induction of LTP. The blue lines refer to the power-law fitting to estimate *α*. **(B)** Same experiment and same experimental epochs as in panel **(A)** analyzed by the AF. Line colors and notation as in panel **(A)**. In both panels **(A)** and **(B)**, a very clear slope change is detected following LTP induction. The AF plot is normalized for the value at Tau = 1 s. **(C)** Bars plot the average estimates for *α* measured with both PG and AF methods before (white bars) and after (black bars) the induction of LTP (*n* = 16 experiments; before *α*_PG_ = 0.11 ± 0.03, LTP *α*_PG_ = 0.3 ± 0.08, **p < 0.01; before *α*_AF_ = 0.083 ± 0.028, LTP *α*_AF_ = 0.316 ± 0.06, ***p < 0.001; mean ± SEM, Wilcoxon signed-rank test, one tail). **(D)** Bars plot the average estimates for *α* measured with both PG and AF methods before (white bars) and after (black bars) the application of the induction protocol in condition known to prevent LTP (postsynaptic BAPTA, extracellular APV) (see text for mini frequency data; *n* = 3 experiments; before *α*_PG_ = −0.034 ± 0.015, LTP-BLOCK *α*_PG_ = 0.144 ± 0.09; before *α*_AF_ = 0.034 ± 0.029, LTP-BLOCK *α*_AF_ = 0.085 ± 0.07; mean ± SEM; *p* > 0.05, Wilcoxon signed-rank test, one tail). **(E)** Values of *α* estimated using PG and AF on individual LTP experiments. White and black circles refer to experiments where we could or could not reject the homogeneous Poisson hypothesis, respectively (PG and AF: *p* < 0.05 for 9/16 cases). Average values (boxes) and error bars are shown on the same plots (LTP *α*_PG_ = 0.3 ± 0.312; LTP *α*_AF_ = 0.316 ± 0.252; mean ± SD; *n* = 16). **(F)** Scatter plot of *α* measured by the AF method vs. the average fold change in mini frequency induced by LTP with linear fitting. Each circle refers to an individual LTP experiment. The fold change in mini frequency was not as broad as with α-LTX (2.4 ± 1.0 fold change; *n* = 16). In these conditions no significant correlation between *α* and LTP change in mini frequency could be extracted (*p* ≥ 0.05, Pearson and Spearman correlation tests).

Following the induction of LTP we tested for a possible increase in the power-law behavior encountered in the control epochs using PG and AF methods (Figures 7A,B). In these experiments, both the incidence and the magnitude of the power-law behavior were significantly increased following LTP induction. Regarding the magnitude of power-law behavior, as summarized in Figure 7C, the value of *α* measured with both PG and AF was significantly increased by LTP induction (*n* = 16; before *α*_P*G*_ = 0.11 ± 0.03, LTP *α*_PG_ = 0.30 ± 0.08, *p* < 0.01; before *α*_AF_ = 0.08 ± 0.03, LTP *α*_AF_ = 0.32 ± 0.06, *p* < 0.001; mean ± SEM; Wilcoxon signed-rank test, one tail). LTP is known to be initiated by Ca^2+^ entry through postsynaptic NMDA receptor channels (Bliss and Collingridge, 2013). To determine if the potentiation in mini frequency required Ca^2+^ entry and Ca^2+^ accumulation in the postsynaptic cell, we run experiments where LTP induction was attempted in cells loaded postsynaptically with the Ca^2+^-chelator BAPTA (BAPTA, 10 mM; APV, 25 µM). In these conditions, following the application of the high K^+^ protocol, after a brief transient change in mini frequency, which subsided 5–10 min after induction, no long-lasting increase in mini frequency was observed (*n* = 3 cells; *p* = 0.25; Wilcoxon signed-rank test, one tail). While the analysis of power-law behavior in the control LTP experiments showed a significant increase in *α*, in the BAPTA sample no significant increase in *α* could be revealed by 5–10 min from the induction protocol either by PG or AF (Figure 7D; before *α*_PG_ = −0.03 ± 0.01, LTP-BLOCK *α*_PG_ = 0.14 ± 0.09, *p* = 0.13; before *α*_AF_ = 0.03 ± 0.03, LTP-BLOCK *α*_AF_ = 0.08 ± 0.07, *p* = 0.38; *n* = 3; mean ± SEM; Wilcoxon signed-rank test, one tail).

Concerning the incidence of power-law in quantal release series following the successful induction of LTP, after induction, 9 out of 16 of LTP experiments were found above the 95% confidence limit with both PG and AF methods (Figure 7E). Comparing these results with those obtained with α-LTX, the fold change in mini frequency following LTP induction was not as broadly distributed (1.3–4.9 fold change). Because of this and possibly because of a concomitant increase in *N*, no significant correlation between *α* and the frequency change could be detected from the LTP experiments (Figure 7F; *p* ≥ 0.05 for Pearson and Spearman correlation tests).

## Discussion

Here we have provided some novel experimental evidence that can be used to explain the power-law behavior of quantal release previously described at hippocampal synapses in single-synapse and population recordings (Lowen et al., 1997; Lamanna et al., 2011). In these experiments, we confirmed the presence of this behavior. We could not reject the homogeneous Poisson hypothesis in most control experiments while a very clear power-law behavior followed the application of α-LTX, a neurotoxin known to greatly enhance spontaneous quantal exocytosis by acting at the very last step of exocytosis (Capogna et al., 1996; Südhof, 2001). Therefore our results clearly suggest that the 1/f behavior must reflect a process occurring upstream exocytosis, unmasked in the high *p* α-LTX condition. The latter scenario was confirmed by computer simulations where the synaptic output was computed by mimicking the discharge of quanta from multiple and independent release sites filled of docking vesicles by an upstream, 1/f source. We also found that the induction of LTP, which produced a long-lasting increase in the frequency of miniature events, significantly enhanced the power-law behavior of spontaneous quantal releases. Based on the unchanged amplitude and kinetics of miniature events after LTP induction, a result speaking against the postsynaptic potentiation of distal or very weak units, we can conclude that LTP increases the contribution of high *p* release sites to the recorded quantal rate. This might be produced either by an increase in *p* at active terminals or by the selective recruitment of high-*p* postsynaptically silent units.

### Temporal dynamics of quantal release

The motivation of this work came from the consideration that an element, often neglected, which must contain valuable information on the state of the presynaptic compartment, concerns the dynamics of spontaneous quantal exocytosis. According to the homogeneous Poisson hypothesis, the generation of minis arises from some latent random process occurring in the presynaptic terminal at very low rate. Such a low rate of discharge should mask or filter the temporal dynamics of all upstream and downstream phenomena, supposedly many orders of magnitude faster. This type of random process should generate uncorrelated quanta and this prediction holds even when changes in the mean rate occur or when the contribution of a population of synapses with distinct Poisson rates is summed. At hippocampal synapses, some evidence for deviations of spontaneous release from the homogeneous Poisson hypothesis has been reported (Lowen et al., 1997; Abenavoli et al., 2002). Indeed, the sequential order of minis could provide information about a multitude of dynamical processes occurring at different time scales inside the presynaptic terminal, which are likely to produce various forms of correlation among the observed quanta. Our results confirm previous evidence for the presence of power-law behavior in quantal release series (Lowen et al., 1997; Takeda et al., 1999; Leao et al., 2005; Lamanna et al., 2011), an interesting feature since this can potentially reveal the existence of long-term correlations (also referred to as long-range dependance, LRD) in the spontaneous release process (Lowen et al., 1997). In most of our control experiments this feature was below the level of significance (Figure 2G), but it was fully revealed when release probability was augmented by α-LTX (Figure 4). This suggests that in high-*p* conditions dynamics upstream vesicular binding to docking sites start contributing to the timing of spontaneous releases.

### Power-law behavior and long-term synaptic plasticity

Long-term synaptic plasticity is a fundamental element for information processing and storage in neuronal circuits (Bliss and Lomo, 1973; Bliss and Collingridge, 2013). Results presented in Figures 6, 7 show that after LTP induction the ~200% increase in mini frequency is accompanied by a significant enhancement of power-law behavior in sequences of miniatures. Since the dynamics of quantal release reflect events occurring in the presynaptic environment and since the appearance of a power-law behavior correlate with an increase in *p*, we can conclude that our results support the idea that LTP must be at least in part sustained by an increase in release probability. This might occur at already functional units (Malgaroli et al., 1995; Ryan et al., 1996; Emptage et al., 2003; Bayazitov et al., 2007; Ratnayaka et al., 2012) and/or might involve the selective recruitment of previously silent synapses (Isaac et al., 1995; Liao et al., 1995), which in any case must be characterized by a higher release probability when compared with previously active units.

Regarding the physiological relevance of this process, despite 60 years have passed since the discovery of minis, a complete and general understanding of their regulation and physiological role is not available yet. Along these years important evidence has accumulated in favor of the role of minis in many different synaptic processes including plasticity (Malgaroli and Tsien, 1992; McKinney et al., 1999; Tyler and Pozzo-Miller, 2003; Espinosa and Kavalali, 2009; Jin et al., 2012; Turrigiano, 2012; Choi et al., 2014). Since central synapses contain few release units, depletion of release sites by diverse fractal regimes of spontaneous exocytosis would affect the synaptic reliability as well as its plastic potential. In this respect, long-term correlations might simply echo the phenotype of plastic changes occurring in the presynaptic compartment. Alternatively, the modulation and/or unmasking of these fractal features might represent a communication channel for the presynaptic compartment to inform the postsynaptic neuron about its state (e.g., its release probability), even when the average release rate is scaled up or down by other homeostatic phenomena (Pozo and Goda, 2010; Turrigiano, 2012).

### Some hypothesis about the emergence of synaptic power-law behavior

**Figure 8:**
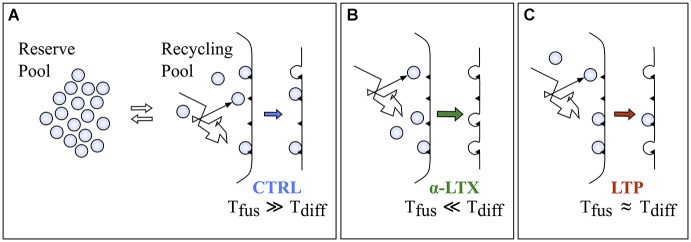
**A possible model to explain the increment in the *α* exponent following α-LTX and LTP. (A)** A cartoon representing the present model of synaptic vesicle exocytosis with a reserve and a recycling pool of vesicles. Black triangles on the membrane refer to docking or release sites (*N*). In this model recycling vesicles should freely diffuse in the cytosol until they bind to docking sites to be released (*T*_diff_ is the average time to reach the docking site). Spontaneous fusion would occur after a random period of residence at the docking sites (*T*_fus_ is the average time to have the release of a docked vesicle). If in control conditions release probability is very low then *T*_fus_ ≫ *T*_diff_, then the Brownian dynamics of recycling vesicles diffusion inside the synaptic cytosol would not sensibly affect the timing of spontaneous exocytosis. **(B,C)** Conditions increasing release probability to high (panel **B**, α-LTX) or intermediate (panel **C**, LTP) levels would make the transfer of the 1/f dynamics more efficient because *T*_fus_ would approach or even go below the value of *T*_diff_.

The occurrence of power-law behavior has been reported in many different physiological contexts, although often a mechanistic understanding is missing. Importantly, the observation of straight lines on log-log scales, the expected appearance of power-laws, *per se* is not sufficient to conclude that the underlying process is indeed 1/f. Other phenomena, including multi-exponentials, in some conditions could mimic the appearance of power-law (Chu-Shore et al., 2010). It would be therefore important to propose possible mechanistic hypothesis to explain the generation of the power-law structure, to better support such a model. The divergence of spontaneous quanta generation from the prediction of the homogeneous Poisson hypothesis, confirmed by two descriptors (PG and AF) working on different domains, clearly suggests that the source of this divergence is inside the exocytotic apparatus. If all events occurring inside the synapse leading to quantal release are lumped together in two sequential steps, a pre-docking and a fusion step (Figure 8), the observed divergence might involve either one of them. The here reported power-law enhancement by α-LTX (Figure 4), known to act at the very last step of fusion, strongly suggests that the source of this divergence is likely to act on the pre-docking step. Among possible sources, the simplest hypothesis relates to the Brownian diffusion of some key element inside the presynaptic compartment. Brownian diffusion is indeed known to generate a fractal-like output in many different contexts (Sokolov and Klafter, 2005). But what could be diffusing? One likely candidate is the synaptic vesicle. Vesicles belonging to the recycling compartment must diffuse in the synaptic milieu to reach their docking sites. An alternative might relate to the complex dynamics of binding and diffusion of calcium ions inside nerve terminals (Zucker and Regehr, 2002; Augustine et al., 2003). These might affect either the recycling pool or the binding affinities of docking sites (Neher and Sakaba, 2008; Vyleta and Smith, 2011). In the past in the same system we showed that the rate of spontaneous exocytosis is poorly dependent on extracellular and intracellular calcium (Abenavoli et al., 2002) and this even during the maintenance of LTP (Malgaroli and Tsien, 1992). Also, following α-LTX application, we observed 1/f behavior even when intracellular calcium was clamped to very low levels, suggesting that these signaling mechanisms are not essential for its generation (unpublished data). Therefore, the most simple and conservative hypothesis relates to the unmasking, in high *p* conditions, of synaptic vesicle Brownian diffusion, a process that must be characterized by 1/f-like dynamics. This process would not affect control spontaneous release statistics because of the very low intrinsic spontaneous release probability (Figure 2), with docked vesicles resting for very long times at the active zone. As schematized in Figure 8, recycling vesicles diffuse in the cytosol until they dock to release sites (*T*_diff_). After a variable waiting time they then undergo spontaneous fusion (*T*_fus_). Since in control conditions vesicle fusion occurs at very low rate (*T*_fus_ ≫ *T*_diff_), Brownian dynamics of diffusing recycling vesicles would not sensibly shape the timing of spontaneous exocytosis. On the contrary, in conditions of high release probability these dynamics would transfer more effectively into the quantal release rate, which would therefore deviate from the prediction of a homogeneous Poisson process. Despite some recent reports have begun providing precise estimates about the diffusional dynamics of synaptic vesicles inside *boutons* (Peng et al., 2012), the evaluation of our hypothesis and its formal demonstration would certainly require additional and more direct experimental evidence. In any case minis are a powerful source of electrical variability or noise for the postsynaptic cell, and as for other forms of noise (Moreno-Bote, 2014) their effect should propagate at all scales. Because of this, the power-law dynamics of synaptic vesicles would be expected to transfer at least in part in neuronal spike series up to network oscillations.

In conclusion, we propose that power-law behavior seen in quantal release series arises from a pre-docking source with a power-law distributed output. Therefore, 1/f behavior could be used as an additional tool to evaluate the state of the release machinery, i.e., its release probability. Since this tool is independent on the number of synapses and release sites, this could be effectively used as a functional state marker of synapses, capable of reporting the previous history of presynaptic *boutons* more directly than the mini frequency *per se*.

## Author contributions

JL performed the experiments and the data analysis; JL and AM designed the study; SC and MGS contributed to the analysis procedures; all authors contributed writing the paper.

## Conflict of interest statement

The authors declare that the research was conducted in the absence of any commercial or financial relationships that could be construed as a potential conflict of interest.
